# Effect of dietary supplementation on the prognostic value of urinary and serum 8-isoprostaglandin F2_α _in chemically-induced mammary carcinogenesis in the rat

**DOI:** 10.1186/1476-511X-10-40

**Published:** 2011-03-03

**Authors:** Barbara Bobrowska, Andrzej Tokarz, Sławomir Białek, Małgorzata Seweryn

**Affiliations:** 1 Department of Bromatology, Medical University of Warsaw, Poland, Banacha 1, 02-097 Warsaw; 2 Department of Biochemistry and Clinical Chemistry, Medical University of Warsaw, Poland, Banacha 1, 02-097 Warsaw

## Abstract

**Backround:**

The aim of the present study was to assess the effects of zinc or copper and polyphenolic compounds on the 8-isoprostaglandin F_2α _concentration in the serum and urine of rats with mammary cancer (*adenocarcinoma*) induced with 7,12-dimethylbenz[a]antracene. The research focused on the kinetics of alterations in urinary 8-isoPGF_2α _at the early stage of carcinogenesis as well as the influence of dietary factors on the process. The impact of selected compounds on the intensity of DMBA - induced carcinogenesis was also assessed.

**Result and conclusions:**

Administration of DMBA, a compound that inducers mammary tumors in experimental animals, increased the serum and urinary 8-isoPGF_2α _levels in study rats. In the rat model, diet supplementation with zinc, combined with selected polyphenolic compounds (resveratrol or genistein) yielded a statistically significant decrease in the rat serum and urinary biomarker concentration with a simultaneously significant stimulation of carcinogenesis.

The results indicate that there is an inverse correlation between the intensity of DMBA-induced carcinogenicity and the level of 8-isoPGF_2α _in urine and serum of rats.

## Introduction

Breast cancer represents the most common neoplastic disease in females, accounting for up to one third of new diagnoses of women's cancer in certain region of the world. The fundamental issue in breast cancer control is prevention, which depends on detection of the determinants of the disease, in terms of initiation and promotion. It is worth mentioning that despite numerous research conducted in different areas, currently there is no one ideal, specific biomarker of the early phase of cancer development, including breast cancer. The possibility of using biomarkers of breast carcinogenesis, such as 8-isoprostaglandin F_2α_, and dietary factors is very promising [1,2].

F_2_-isoprostanes are the isomers of F-prostaglandin, produced by cyclooxygenase-independent free radical peroxidation of arachidonic acid. The formation of 8-isoPGF_2α _causes damage to biomembranes and to oxidative modification of plasma lipoproteins. Carcinogenesis may be initiated by lipid peroxidation, which ultimately produces damage to proteins and DNA [3].

Another problem is the influence of dietary factor on biomarkers and the mechanisms of the disease development process. It is estimated that approximately one-third of human cancers are associated with inappropriate dietary habits and lifestyle. Therefore, dietary intake of trace elements and polyphenols becomes an important factor in controlling oxidative stress and the pathologies that ensue [4-8].

The aim of the present study was to assess the effect of zinc or copper and polyphenolic compounds (resveratrol or genistein) on the concentration of 8-isoprostaglandin F_2α _in the serum and urine of rats with mammary cancer (*adenocarcinoma*) induced with 7,12-dimethylbenz[a]antracene. No reports have shown kinetic alterations in the urinary 8-isoPGF_2α _quantity excreted at the early stage of carcinogenesis or the impact of dietary factors on the process. The impact of selected compounds on the intensity of DMBA - induced carcinogenesis was also assessed.

It is still not clear what kind of combinations and doses of trace elements and polyphenolic compounds should be given in order to achieve the most beneficial health effects in human cancer prevention or for improvement of pharmacological treatment.

## Materials and methods

### Laboratory animals

Female Sprague-Dawley rats were obtained from the Animal Laboratory, Department of General and Experimental Pathology, Medical University of Warsaw. The study was approved by the Ethics Committee, Medical University of Warsaw. The animals were divided into 12 groups (Table 1). Those in groups 1 to 6 (n = 60) were treated twice with of 80 mg/kg of body weight DMBA (Sigma-Aldrich, St. Louis, MO, USA) in rapeseed oil (via gavage) to induce mammary cancer (*adenocarcinoma*); the first treatment was given at 50 days, followed by the same dose (a repeat dose of 80 mg/kg body weight) at 80 days of rat age. Rats from groups 7 to 12 (control groups) (n = 40) were accommodated under the same conditions as those from groups 1 to 6, and received the same diet, but no DMBA treatment. Tumors were evaluated histopathologically to confirm malignancy and to prove that they were *adenocarcinomas *(II and III degree). In DMBA-untreated groups spontaneous cancers were not observed.

**Table 1:** Composition of diets administered of different groups

Diets	groups/carcinogens											
	
	DMBA +						DMBA -					
	
	1	2	3	4	5	6	7	8	9	10	11	12
1. Standard diet	+	+	+	+	+	+	+	+	+	+	+	+
2. Zinc 6.9 mg/mL (231 mg Zn/kg diet)	-	+	-	-	-	-	-	+	-	-	-	-
3. Zinc 6.9 mg/mL (231 mg Zn/kg diet) and resveratrol 0.1 mg/mL (0.2 mg/kg bw)	-	-	+	-	-	-	-	-	+	-	-	-
4. Zinc (6.9 mg/mL) (231 mg Zn/kg diet) and genistein 0.1 mg/mL (0.2 mg/kg bw)	-	-	-	+	-	-	-	-	-	+	-	-
5. Copper 1.3 mg/mL (42.6 mg Cu/kg diet)	-	-	-	-	+	-	-	-	-	-	+	-
6. Copper 1.3 mg/mL (42.6 mg Cu/kg diet) and resveratrol 0.1 mg/mL (0.2 mg/kg bw)	-	-	-	-	-	+	-	-	-	-	-	+

Abbreviations: DMBA - 7,12-dimethylbenz[a]anthracene; DMBA (+) corresponds to DMBA-treated groups; DMBA (-) corresponds to DMBA-untreated groups;

All animals had a free access to water and food (standard diet: Labofeed H, Żurawia 19, 89-240 Kcynia, Poland). Table 1 shows additional different types of bioelements or polyphenolic compounds also given to the animals. The rats were fed extra supplements suspended in water, (0.4 ml daily via gavage), from 40 days until 20 weeks of age (sacrifice time by decapitation). The animals were fed only the standard diet (without supplementation) received 0.4 ml of water. The polyphenol dose levels were selected based on human average daily consumption (extrapolating on the rats' body weight) [9,10]. The doses of bioelements were established based on the values used in the Labofeed H diet (standard diet) (77 mg Zn/kg diet; 21.3 mg Cu/kg diet) [11]. The zinc and copper content in the standard diet were determined following wet microwave sample mineralization using atomic absorption spectrophotometry (AAS). The dose levels of each agent were selected for projected drug levels.

In order to obtain rat urine samples, the animals were placed in individual metabolic cages for 24 hours. Urine was collected at 10 and 12 weeks of age, at an early, nonpalpable stage of carcinogenesis. The animals were sacrificed by decapitation at 20 weeks of age, and serum were collected. The biomaterial samples were stored at a temperature of - 70C until the test time.

The concentration of 8-isoPGF_2α _in serum and urine was determined by a competitive enzyme-linked immunosorbent assay (ELISA) (8-isoprostane EIA kit, Cayman Chemical Company (Ann Arbor. MF, USA).

### Determination of creatinine level

The 8-isoPGF_2α _level was standardised by conversion to the creatinine level. The latter was examined in urine samples with creatinine test (Hydrex, Warsaw, Poland) based on Jaffe's reaction (in an alkaline environment creatinine forms a colored complex with picric acid).

### Superoxide dismutase (SOD) activity

Superoxide dismutase activity in serum was determined with the Superoxide Dismutase Assay kit, Cayman Chemical Company (Ann Arbor, MI). The assay kit utilizes a tetrazolium salt for detection of superoxide radicals generated by xanthine oxidase and hypoxanthine. One unit of SOD is defined as the amount of enzyme needed to exhibit 50% dismutation of the superoxide radical. The SOD assay measures all three types of SOD (Cu/Zn, Mn and FeSOD).

The animals were examined by palpation during the study to characterize the time course of tumor development. At sacrifice (at 20 weeks of rats's age) tumors were removed, weighed and histopathological examinaton was performed.

The results obtained were statistically assessed using the Kolmogorow-Smirnow test, ANOVA with POST-HOC NIR tests (SPSS 12 programme).

## Results and discussion

**Figure 1:**
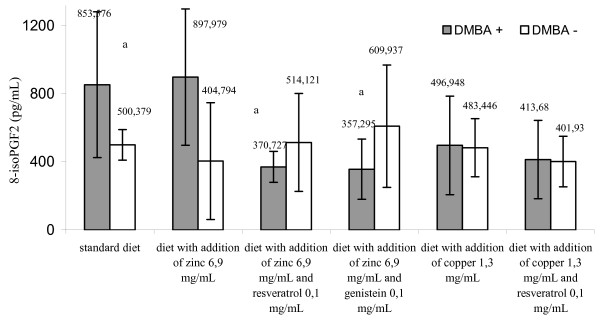
**The content of 8-isoPGF_2α _(pg/mL) (means ± standard deviation) in serum of DMBA treated and untreated animals in relation to diet**. a - p < 0.05 group treated DMBA, fed standard diet versus groups treated DMBA, fed diet with addition of zinc or copper separately or in combination with resveratrol and genistein. The date refer to 20 weeks of the animals' age (decapitation time). Abbreviations: DMBA - 7,12-dimethylbenz[a]anthracene; DMBA (+) corresponds to DMBA-treated groups; DMBA (-) corresponds to DMBA-untreated groups; 8-isoPGF_2α _- 8-isoprostaglandin F_2α_.

**Figure 2:**
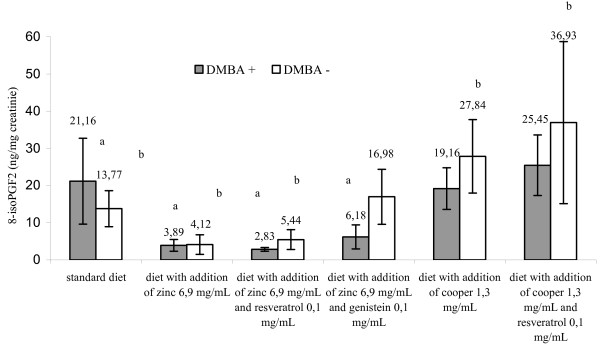
**Urinary 8-isoPGF_2α _(ng/mg creatinine) in rats fed different diets in early-onset breast cancer - 10 week of the rodent's age**. a - p < 0.05 group treated DMBA, fed standard diet versus groups treated DMBA, fed diet with addition of zinc or copper separately or in combination with resveratrol and genistein. b - p < 0.05 group untreated DMBA, fed standard diet versus groups untreated DMBA, fed diet with addition of zinc or copper separately or in combination with resveratrol and genistein. Abbreviations: DMBA - 7,12-dimethylbenz[a]anthracene; DMBA (+) corresponds to DMBA-treated groups; DMBA (-) corresponds to DMBA-untreated groups; 8-isoPGF_2α _- 8-isoprostaglandin F_2α_.

**Figure 3:**
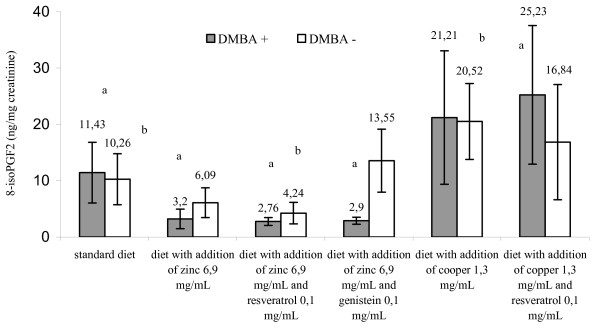
**Urinary 8-isoPGF_2α _(ng/mg creatinine) in rats fed different diets in early-onset breast cancer - 12 week of the rodent's age**. a - p < 0.05 group treated DMBA, fed standard diet versus groups treated DMBA, fed diet with addition of zinc or copper separately or in combination with resveratrol and genistein. b - p < 0.05 group untreated DMBA, fed standard diet versus groups untreated DMBA, fed diet with addition of zinc or copper separately or in combination with resveratrol and genistein. Abbreviations: DMBA - 7,12-dimethylbenz[a]anthracene; DMBA (+) corresponds to DMBA-treated groups; DMBA (-) corresponds to DMBA-untreated groups; 8-isoPGF_2α _- 8-isoprostaglandin F_2α_.

Mammary glands of several rat strains, mainly Spraque-Dawley, are susceptible to transformation induced by chemical carcinogens. One widely used active chemical inductors of mammary carcinogenesis is 7,12-dimethylbenz[a]anthracene [1]. The results of this study indicated that the amount of 8-isoPGF_2α _in serum and urines from rats treated with cancerogenic factor and receiving a standard diet was higher than in the control group receiving analogous diet but with no DMBA treatment, however, the differences were not statistically significant for the reason of high values of standard deviation (Figures 1, 2 and 3). Similar trend regarding amount of 8-isoPGF_2α _in serum of rats treated with carbon tetrachloride was acquired by Kadiiska et al. [12]. Administration of DMBA resulted in an increase of biomarkers indicating damage of DNA (8-hydroxy-2'-deoxyguanosine), proteins (carbonyl groups of amino acids) and lipids (8-isoPGF_2α_) [13]. These findings may confirm the relationship between DMBA and stimulation of the free radical processes.

It was found that zinc administration combined with selected polyphenolic compounds (resveratrol or genistein) statistically decreased the biomarker content level in the rat serum and urine compared with the animals receiving exclusively a standard diet (p < 0,05) (Figures 1, 2 and 3). It was demonstrated that diet supplementation with zinc did not decrease the content level of the studied biomarker in serum of DMBA-treated rats. On the contrary, it stimulated the process (853,576 ± 428,232 versus 897,979 ± 400,809 pg/ml), although, the response was not statistically significant (p < 0,05). In this case, synergistic reactions between Zn and polyphenols was observed. Evidence shows an ability of polyphnols to scavange free radicals, build complexes with metal ions catalysing formation of free radicals, and also inhibiting certain enzymes participating in oxidation [6-8].

Zn plays an essential role in cell membrane integrity and is a component of more than 300 different enzymes that function in many aspects of cellular metabolism. In numerous systems Zn can antagonize the catalytic properties of the redox-active transition metals (Fe and Cu) with respect to their abilities to promote formation of hydroxyl radical (^.^OH) from H_2_O_2 _and superoxide [14]. Surprisingly, the results of our work indicated no connection between superoxide dismutase activity and Zn or Cu supplemented diets (Table 2). Therefore, the decision was made to discontinue the search for mutual correlations of 8-isoPGF_2α _concentration, dependent on the diet and the enzyme activity, especially, that the rat group receiving additional zinc exhibited a stimulated lipid peroxidation (Table 2, Figures 1, 2 and 3). There is no univocal evidence in the literature to confirm that dietary zinc supplementation in applied dose exerts an impact on the activity of superoxide dismutase. Reports seem to indicate that diets supplemented with zinc in different quantities did not cause significant changes in SOD activity, what was also confirmed by our study [15,16].

**Table 2:** Superoxide dismutase activity in rat's serum in relation to diet

diet	Cancerogenic factor	Superoxide dismutase (U/mL) activity in serum
1. Standard diet	DMBA +	19.73 ± 2.82
	DMBA -	22.34 ± 1.65
2. Zinc 6.9 mg/mL (231 mg Zn/kg diet)	DMBA +	19.52 ± 1.83
	DMBA -	19,11 ± 3,77
3. Zinc 6.9 mg/mL (231 mg Zn/kg diet) and resveratrol 0.1 mg/mL (0.2 mg/kg bw)	DMBA +	20.81 ± 2.02
	DMBA -	21.96 ± 2.31
4. Zinc (6.9 mg/mL) (231 mg Zn/kg diet) and genistein 0.1 mg/mL (0.2 mg/kg bw)	DMBA +	19.69 ± 2.67
	DMBA -	21.93 ± 2.27
5. Copper 1.3 mg/mL (42.6 mg Cu/kg diet)	DMBA +	20.64 ± 2.24
	DMBA -	22.29 ± 3.99
6. Copper 1.3 mg/mL (42.6 mg Cu/kg diet) and resveratrol 0.1 mg/mL (0.2 mg/kg bw)	DMBA +	19.74 ± 3.20
	DMBA -	22.07 ± 4.01

The date refer to serum evaluated at 20 weeks of the animals' age (decapitation time)

Abbreviations: DMBA - 7,12-dimethylbenz[a]anthracene; DMBA (+) corresponds to DMBA-treated groups; DMBA (-) corresponds to DMBA-untreated groups;

Many reasearchers have investigated the dietary supplementation effects on the levels of urinary isoprostane or isoprostane metabolites. Diets rich in fruit and vegetables diminish the excretion of urinary 8-isoPGF_2α _[2]. The streptozotocin model of diabetes used in the rats showed reduced biomarker levels following the diet supplementation with vitamin E [17]. Urinary and plasma F_2_-isoprostanes were significantly decreased with alcohol-free red wine [18].

Cooper is generally considered as a pro-oxidant metal that participates in Fenon-like reactions. One of the mechanisms of procancerous Cu activity involves its ability to produce a strongly reactive hydroxyl radical responsible for oxidative damage to DNA strands and also peroxidation of cell membrane lipids [14]. As shown in our paper, the animals receiving diet supplemented with copper or copper and resveratrol had significantly higher amount of 8-isoPGF_2α _in urine compared with the animals receiving a standard or zinc supplemented diet (p < 0,05) (Figure 2, 3). This implies a pro-oxidant effect of cooper.

Another relevant issue in our study was to assess the severity of carcinogenesis expressed by tumor weight and number in particular rat groups as well as the onset of the initial tumors (Table 3). The data refer to tumors evaluated in 20th week of the animals age (decapitation time). The largest and most numerous tumors per animal were found in the group receiving zinc and resveratrol supplemented diet, which might have been due to the fact that in that group the first tumors appeared at 13 weeks of rat age, i.e. two weeks earlier than in the group fed the standard diet. Zinc compounds are considered to be chemopreventive agents chiefly due to their antioxidative properties. However, reports show that excessive Zn in the animal body may exacerbate tumorigenesis [14]. The harmful role of Zn in tumorigenesis could depend on the dose of zinc and synergism between various antioxidant used in the study. What is interesting there was an inverse correlation between the intensity of cancer development process and the level of 8-isoPGF_2α _in urine and serum of rats. A plausible explanation of this fact could be that Zn acts to improve health through a wide variety of biological mechanisms and not antioxidant activity alone. Thomson et al [19] reported a moderately significant inverse association of 8-isoPGF_2α _within plasma carotenoid levels, a protective association not seen with dietary carotenoid levels.

**Table 3:** Tumor induction in 7.12-dimethylbenz[a]antracene treated groups in relation to diet

Diet	Tumor incidence (%)^1^	Number of tumors in one rat	Maximum tumor weight in one rat in group (g)	First week at onset
1. Standard diet	9/9 (100)	1-5	12	15
2. Zinc 6.9 mg/mL (231 mg Zn/kg diet)	9/9 (100)	1-5	20	15
3. Zinc 6.9 mg/mL (231 mg Zn/kg diet) and resveratrol 0.1 mg/mL (0.2 mg/kg bw)	10/10 (100)	2-10	30	13
4. Zinc (6.9 mg/mL) (231 mg Zn/kg diet) and genistein 0.1 mg/mL (0.2 mg/kg bw)	10/11 (91)	1-6	15	15
5. Copper 1.3 mg/mL (42.6 mg Cu/kg diet)	9/9 (100)	1-5	7	14
6. Copper 1.3 mg/mL (42.6 mg Cu/kg diet) and resveratrol 0.1 mg/mL (0.2 mg/kg bw)	12/12 (100)	2-6	11	14

The date refer to tumors evaluated at 20 weeks of the animals' age (decapitation time).

^1 ^Tumor incidence is expressed as the number of tumor-bearing rats over total number of rats in the group.

## Conclusions

Administration of DMBA, a compound that induces mammary cancer, increased the level of 8-isoPGF_2α _in serum and urine of tested rats. In this model, a diet supplementation with zinc combined with selected polyphenolic compounds (resveratrol or genistein) caused statistically significant decrease in concentration of the studied biomarker (8-isoPGF_2α_) in rat serum and urine with simultaneous significant stimulation of carcinogenesis. Finally, the level of 8-isoPGF_2α _seen in the serum and urine in the rat model did not indicate a positive correlation with the risk of carcinogenesis, and this response, to some extent negates the value of 8-isoPGF_2α _as a potential determinant of early pathogenesis of cancer.

## List of abbreviations

AAS: atomic absorption spectrophotometry, Cu: copper, DMBA: 7,12-dimethylbenz[a]anthracene, ELISA: enzyme-linked immunosorbent assay, SOD: superoxide dismutase, Zn: zinc, 8-isoPGF_2α_: 8-isoprostaglandin F_2α_.

## Competing interests

The authors declare that they have no competing interests.

## Authors' contributions

BB planed, designed and carried out the expeiment and performed the statistical analysis. AT coordinated the study. SB and MS carried out the ELISA analysis. All authors read and approved the final manuscript.
